
JOURNAL INFORMATION
==============================
NLM Title Abbreviation: Tex Med J (Austin)
NLM Journal ID: 21130590R

Texas Medical Journal

ISSN: 0892-8495
Publisher: Biomedical Journal Digitization Project

ARTICLE INFORMATION
==============================
PMCID: PMC9590758
Article version: 1
Subjects: Books and Magazines

Essentials of Bacteriology

Being a Concise and Systematic Introduction to the Study of Micro-Organisms for the Use of Students and Practitioners

Print publication date: 1898 Sep
Volume: 14
Issue: 3
First page: 155
Last page: 155
License: This work is free of known copyright restrictions. For more information, please see PMC Back Issue Digitization.
License URL: https://creativecommons.org/publicdomain/mark/1.0/

==============================

### For Sale.

**For Sale.**—A good country practice,—good collections, and in good farming country. Cost of property will secure practice. A good thing for the right party. Address,

E. C. Partee, M. D., Laughlin, Ark.

**For Sale.**—Half interest in an office practice that pays Seven Thousand Dollars a year, cash. Address, Box 117, Dallas, Texas.

### Books and Magazines.

Essentials of Bacteriology. Being a Concise and Systematic Introduction to the Study of Micro-organisms for the use of Students and Practitioners. By M. V. Ball, M. D., Bacteriologist to St. Agnes' Hospital, Philadelphia. Third Edition, Revised. With Eighty-one Illustrations, some in Colors, and Five Plates. Philadelphia: W. B. Saunders, 925 Walnut Street. 1897. Price, $1.

This compend has been received with so much favor by the profession that a third edition became necessary. The arrangement of this volume is particularly suitable for medical students, furnishing as it does a laboratory hand-book and a systematic introduction into the science of bacteriology.

In this edition, the chapter on diphtheria has been carefully revised, and a chapter on "bacteriologic examinations of the organs and cavities of the human body" has been added to the appendix. It is a very satisfactory elementary work on the subjects considered, and will no doubt continue to receive the full share of favors which the former editions have, and which this one abundantly merits. S. E. H.
